# A scoping review on integrated health campaigns for immunization in low- and middle-income countries

**DOI:** 10.1093/heapol/czad082

**Published:** 2023-09-12

**Authors:** Syeda Tahmina Ahmed, Shams Shabab Haider, Suhi Hanif, Humayra Binte Anwar, Saima Mehjabeen, Svea Closser, Eva Bazant, Malabika Sarker

**Affiliations:** BRAC James P Grant School of Public Health, BRAC University, 6th Floor, Medona Tower, 28 Mohakhali Commercial Area, Bir Uttom A K Khandakar Road, Dhaka 1213, Bangladesh; BRAC James P Grant School of Public Health, BRAC University, 6th Floor, Medona Tower, 28 Mohakhali Commercial Area, Bir Uttom A K Khandakar Road, Dhaka 1213, Bangladesh; BRAC James P Grant School of Public Health, BRAC University, 6th Floor, Medona Tower, 28 Mohakhali Commercial Area, Bir Uttom A K Khandakar Road, Dhaka 1213, Bangladesh; BRAC James P Grant School of Public Health, BRAC University, 6th Floor, Medona Tower, 28 Mohakhali Commercial Area, Bir Uttom A K Khandakar Road, Dhaka 1213, Bangladesh; BRAC James P Grant School of Public Health, BRAC University, 6th Floor, Medona Tower, 28 Mohakhali Commercial Area, Bir Uttom A K Khandakar Road, Dhaka 1213, Bangladesh; John Hopkins University, Bultimore, Maryland 21218, US; The Task Force for Global Health, 330 W. Ponce de Leon Ave., Decatur, GA 30030, US; BRAC James P Grant School of Public Health, BRAC University, 6th Floor, Medona Tower, 28 Mohakhali Commercial Area, Bir Uttom A K Khandakar Road, Dhaka 1213, Bangladesh

**Keywords:** Integrated health campaign, immunization, integration, service delivery, implementation, low- and middle-income countries, scoping review

## Abstract

Health campaign integration is a key implementation strategy outlined by the World Health Organization to achieve universal health coverage. This scoping review synthesizes the evidence on Integrated Health Campaigns (IHC) in the field of immunization in low- and middle-income countries (LMICs) regarding the most common strategies, facilitators and barriers. Four reviewers followed a systematic approach to identify, screen and analyse relevant articles. The team used three search engines (PubMed, Scopus and Google Scholar) to identify peer-reviewed journal articles as well as select institutional websites for grey literature publications. Full-text articles using any study design and across any time frame were included. Data were extracted following a predefined matrix, analysed deductively and presented in a narrative synthesis. Thirty articles (20 academic and 10 grey) were included in the final review. All studies included identified IHCs as effective when planning or implementation is integrated. The common strategies were: using resources efficiently in remote locations; using national immunization days to maximize impact; targeting specific age groups by selecting intervention sites that are frequented by that age group; building community ownership over the integrated program; and integrating programs that already share common elements. The key facilitators were: closing the gap between services and communities; planning, coordination and resource management both before and during integration; cost-effectiveness; and utilization of pre-existing infrastructure. The common barriers included seemingly optimized initial cost to appear feasible only in the short term and additional responsibilities on the field staff. This review finds IHCs a common practice in immunization and identifies gaps in evidence on evaluation; indicating the need for additional research. Strong evidence accounts IHCs to increase coverage, improve community acceptance of health services and strengthen the community models of health service delivery.

Key messages

- This scoping review of literature from LMICs across the world has summarized a collection of common strategies adopted or approaches towards implementation of IHCs in immunization; leveraging mass campaigns like NIDs or NVWs, to popular integrating component, such as, vitamin A supplementations, integrating new/less familiar health intervention in remote areas, choosing implementation site focusing on approaching a specific target population or grouping complementary health services/interventions of a public health issue, or sometimes increasing community involvement often times contributing to the greater good of the intervention.
- Among the facilitators of IHC implementation, a community where health workers had established rapport system, trustworthy relationships and a voice advocating their communities during an immunization campaign; integration of new/low-acceptance services came out as easily incorporable. Similar phenomena was observed in case of an existing infrastructure for immunization campaigns, such as school-based immunization platform, where the stakeholders appeared to take ownership of the implementation efforts.
- The two major barriers to implementation of the IHCs are the cost of integration and the challenge with human resources. According to our review, though cost-effectiveness is deemed as a benefit that comes off integration of health services, it may be only limited to the project/program duration when external funding supports the cost of integration. Unless allocated extra budget exclusively for long-term implementation or scale-up, the IHCs close down after a few years. Our review also identified lack of evidence in comprehensive research focusing on the cost-effectiveness of IHCs. Similarly, for integration of IHCs, a pool of health care providers must be trained, and time and logistics allocated to support implementation of IHCs. Still, activities around integration project an added burden on them alongside their routine responsibilities.

## Introduction

Integrated health campaigns (IHCs) are routinely utilized in the world of implementation, though has not been evaluated for their effectiveness. There is a lack of review of campaign integration practices compared with routine integration practices to date; which calls for this scoping review.

The World Health Organization (WHO) defines integrated health services as ‘health services that are managed and delivered so that people receive a continuum of health promotion, disease prevention, diagnosis, treatment, disease-management, rehabilitation and palliative care services, coordinated across the different levels and sites of care within and beyond the health sector and according to their needs throughout the life course’ (World Health Organization, 2016, page 2). The definition provides direction to the delivery of health services putting people at the centre and suggests integration of vertical services that move away from hospital-based, disease-based and self-contained care models, ensuring continuity of access to various health services for people (WHO, 2016). On the other hand, ‘integrated health campaign’ refers to ‘collaboration’ across the planning, implementation, or evaluation of vertical campaigns which are often program-based and intermittent in nature (Bazant *et al.*, 2022). We consider existing health or immunization campaigns that deliver health services through active surveillance (visiting communities in designated campaign locations following campaign schedules) and integration of another immunization or health campaign across the components of microplanning, financing, logistics, or implementation as ‘Integrated Health Campaign’ (IHC) for this review.

Health campaign integration is one of the strategies used to achieve universal health coverage in low- and middle-income countries (LMICs) (Bright *et al.*, 2017). Routine immunization (RI) has a history of integration with other programs, including Vitamin A supplementation, deworming and insecticide-treated net (ITN) distribution in LMICs (World Health Organization, 2018). The value of integration within immunization programs as well as health systems in general has emerged as the next necessary major step forward in the provision of healthcare on a global scale (World Health Organization, 2018). The Global Vaccine Action Plan 2011–2020 specifically cites ‘integration’ as one of its guiding principles. Adherence to this and other principles will set many countries on the path towards meeting their sustainable development goals (World Health Organization, 2013).

Currently, most health systems in LMICs remain largely fragmented, which is to say there is a lack of coordination and collaboration across various health programs that often have different funding and accountability streams, and integration efforts vary by country and by context (World Health Organization, 2018; The Task Force for Global Health, 2020). Integrated healthcare in LMICs has focused on serving underserved populations and reducing costs (Mounier-Jack *et al.*, 2017), naturally occurring at the service provider level, where various services are provided under a single roof (World Health Organization, 2018; The Task Force for Global Health, 2020).

IHCs are a vital strategy for LMICs to scale up current immunization and other public health campaigns; however, their effects are typically only explored on a case-by-case basis. Our review aims to map the existing evidence on IHCs in the context of immunization in LMICs to better understand how these integration targets are currently being implemented. This is a first step towards better understanding the common facilitators and barriers across all IHCs as well as what strategies or programs may require more attention in future endeavours of implementation and scaling up. The particular research questions asked in this review have been listed below.

### Research questions

This review focused on the following general research question: What is the knowledge of IHCs in the domain of immunization in LMICs?

Our specific research questions included: (1) what evidence is there of various health services being integrated with immunization campaigns? How the components of the health service and the campaign were integrated and to what extent? What is known from the literature on the service delivery outcomes of IHCs, such as increased awareness and improved coverage? (2) What are the facilitators and (3) barriers to the integration of health services with immunization campaigns? (4) What recommendations exist regarding the implementation specifics of IHCs?

Exploring IHCs required the establishment of operational definitions for both integration and health campaigns. In this review,

**Integration** involves sharing, collaboration, and/or coordination of campaign components (e.g., microplanning, registration, logistics, implementation and evaluation) to improve the efficiency and effectiveness of multiple campaigns or allow simultaneous or co-delivery of two or more health interventions at the point of service delivery (Bhatnagar and Gittelman, 2020). This study will only focus on integration where at least one of the campaigns is related to the immunization of humans.

**Health campaigns** are time-bound, intermittent activities deployed to address specific epidemiologic challenges, expediently fill delivery gaps, or provide surge coverage for health interventions (Bhatnagar and Gittelman, 2020, Pg-1). It should be noted that this definition excludes many national programs, which may often integrate multiple components and health services, but reached the population passively through facilities or as a part of routine immunization, not through intermittently scheduled campaigns.

## Method

### The approach of the study

We followed a scoping review approach and used the Preferred Reporting Items for Systematic and Meta-Analyses (PRISMA) Extension for Scoping Reviews (PRISMA-ScR) checklist (Tricco *et al.*, 2018) as a guide to this review which allowed the synthesis of the broadest overview of available literature on a given topic and identify knowledge gaps (Arksey and O’Malley, 2005; Munn *et al.*, 2018). This approach is also useful to identify scoping of a future systematic review and assess the relevance of review questions and eligibility criteria (Munn *et al.*, 2018). World Bank country classifications were followed to include countries in the review as LMICs (World Bank, 2022).

### Eligibility criteria

A detailed scoping review protocol (Table 1) was developed to inform and align all reviewers to the concepts, objectives and eligibility criteria of the review. To formulate the research questions, we utilized the PCC framework (Problem, Content and Context) (Subject Librarian, 2017).

**Table 1.** Scoping review protocol

Objectives	To map the existing evidence on ‘Integrated Health Campaigns’ in the field of immunization from low- and middle-income countries.	
Research question	What is the knowledge on IHCs in the domain of immunization in LMICs?	
Specific research questions	What evidence is there of various health services being integrated with immunization campaigns?	
	How had components of the two services been observed to be integrated and to what extent? What is the effect of IHCs on service delivery outcomes, such as increased awareness and improved coverage?	
	What enabling factors lead to the integration of health services with immunization campaigns? What are the hindering factors that prevent such service delivery or implementation of IHCs?	
	What recommendations were made from implementation experiences of IHCs in the LMICs?	
Search strategy	Inclusion criteria	Full-text peer-reviewed journals from all LMICs, and grey literature regarding Bangladesh specifically, including non-commercially published or unpublished reports, documents, protocols, standard operating procedures, PowerPoint presentations and news articles; on implementation of IHCs, where another vaccine or health service(s) had been integrated with an immunization campaign in an LMIC.
	Exclusion Criteria	Any article with a focus on: – Concept not IHC as per operational definition – Context other than immunization – Country not classified as an LMIC – Any article with data not available/segregated for any of the LMICs
	Language	– English for the overall review – English and Bangla for grey literature focusing on Bangladesh
	Time frame	Any to August 31, 2022
Data source	Peer-reviewed journal and non-academic articles	– PubMed – Scopus – Google Scholar (included non-peer-reviewed articles)
	Grey literature	– Google – Institutional websites of relevant organizations

#### Inclusion criteria

This review included the integration of health services to immunization campaigns that were reaching its catchment population over a few days’ campaign, through door-to-door visits or satellite centres. All academic articles published in peer-reviewed journals that explored IHCs in the field of immunization and implemented in LMICs were included. Additionally, grey literature such as reports, news articles and presentations was included to develop an understanding of the scenario in Bangladesh as a case study, as most of the reviewers were based in Bangladesh thus having more in-depth knowledge about the immunization campaign history in Bangladesh. For academic articles, we sought all study design types (i.e. quantitative, qualitative, reviews, etc.). We limited the language to English for scholarly articles and considered Bengali besides English for grey literature, as the reviewers’ group consisted of bilingual speakers of the two languages. No restriction on time was applied for the start date, but restricted till July 2021 on the first round and updated until August 2022 for searching relevant articles.

#### Exclusion criteria

Any article describing integration to a health campaign in a field other than immunization was not within the scope of the review. Integration to routine immunization or catered at primary, secondary or tertiary health facilities was excluded. Articles with country data not segregated for the LMICs, or lacking reporting of at least one of the LMICs were also excluded from the review. Any article not meeting the inclusion criteria of the concept, language and open access was not included for reviewing.

### Search strategy and identification

We also used the PCC (problem, content and context) framework to identify the themes (P—implementation strategies, challenges, etc.; content—IHC; context—immunization and geographic locations) and compiled a list of synonyms and similar terms for each of the themes (Table 2). We combined the synonyms under each theme and also the thematic areas to tailor the key search terms To ensure the maximum number of relevant articles to be found in the search, we generated one search term for all LMICs in general, two for the regions South Asia and Sub-Saharan Africa and 52 for each of the individual LMICs; summing up 55 search terms in total. The exact key terms are presented for one of the databases (Appendix Table A1). We searched PubMed and Scopus for articles published in peer-reviewed journals and Google Scholar for mixed article types (both peer-reviewed and non-peer-reviewed articles). Search results obtained from Google Scholar were recorded till the first 100 articles; to limit the results to the most relevant articles and ensure the feasibility of the review. Meanwhile, grey literature was searched on the websites of WHO, UNICEF, Rotary International and the Centers for Disease Control and Prevention. All search results and raw files were preserved for future reference. The relevant information for screening was recorded on a Microsoft Excel spreadsheet. The search was conducted over two weeks in the month of July 2021 and updated following the same procedure in August 2022.

**Table 2.** Search strategy

Topic (content) (A)*	Domain (context) (B)	Key outcome (problem) (C)	Geographic location (context) (D)
– Integrated health campaign – Integrated health service – Integrated health project – Integrated health program – Integrated health initiative – Integrated health plan	– Immunization – Vaccination – Vaccines – Inoculation	– History – Evolution – Practice – Implementation – Success factors – Challenges – Barriers – Effectiveness – Evaluation – Lessons learned	– LMICs – LMICs by region—South Asia and Sub-Saharan Africa – LMICs by individual countries

* Broader themes A, B, C, and D have been combined in search terms by using Boolean operator ‘AND’, and titles under each theme have been combined with Boolean operator ‘OR’.

### Selection and evaluation of relevant studies

All search results were compiled in a single Excel file and sorted. Duplicates of records were removed. Next, a two-phase screening process took place. All four reviewers took part in the 1^st^ phase of screening based on the titles and abstracts of the articles. Any indecision regarding the inclusion of an article was resolved by crosschecking and discussion among the reviewers. In the 2^nd^ phase of full-text reviewing and study selection, two reviewers reviewed the inclusion of all articles based on the eligibility criteria. The final decision for articles with a disagreement was resolved with a discussion with a third reviewer.

### Data extraction and analysis plan

Data were extracted for the variables—title, author, journal, year of publication, country, article type, abstract, study objectives, study design/methodology, description of IHC, parameters and main findings under the themes as following: (1) key strategies to IHC, (2) facilitators of implementation, (3) barriers of implementation, (4) recommendations for the health system/ primary health care. The themes were generated as per the requirements of researchers working on a larger mixed-method study to explore Health Campaign effectiveness in various fields, one of which was ‘immunization’, to get a historical overview of the evolution of IHCs and implementation lessons across the globe. The themes were intended to inform a qualitative component to elicit recommendations for IHCs in the case of Bangladesh.

Of the abovementioned themes, ‘facilitators’, ‘barriers’ and ‘recommendations’ were analysed deductively. Data for the theme ‘Key strategies to integrated health campaign’ were organized and sought for patterns, thus analysed inductively.

Three reviewers took part in extraction while a fourth checked all extraction for uniformity and consistency. The extraction process involved the collection of relevant quotes from the included documents and the analysis and arrangement of those quotes based on the identified themes. Data were analysed by one reviewer and checked by another. The review questions to be answered did not require an assessment of the methods applied in the included articles (implementation strategies, facilitators and barriers) and also not typically demanded from a scoping review (Munn *et al.*, 2018). As a result, no critical appraisal of the methods of the studies was conducted, without any effect in synthesizing the findings. Finally, findings were presented in a narrative synthesis. Appendix Table A2 presents all reporting items of the scoping review following the Preferred Reporting Items for Systematic and Meta-Analyses (PRISMA) Extension for Scoping Reviews (PRISMA-ScR) checklist.

## Findings

### Search results and study selection

**Figure 1.**
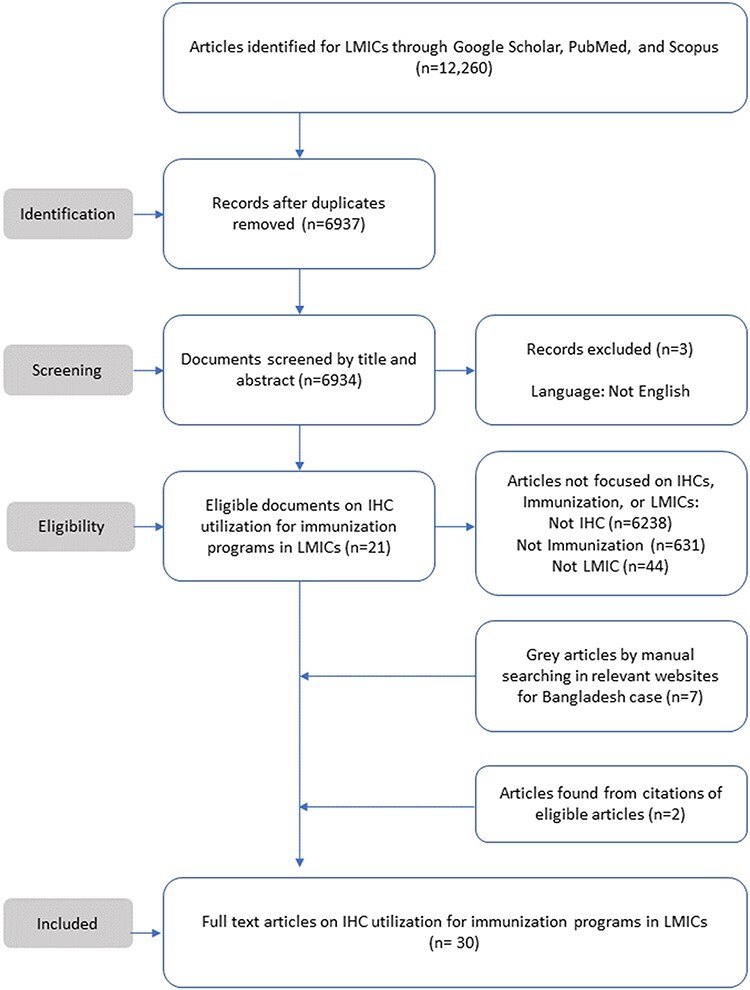
Preferred Reporting Items for Systematic Reviews and Meta-Analyses (PRISMA) flowchart of included studies

The initial search yielded 12 260 documents. Following the removal of duplicates, 6937 documents were marked as potentially relevant for analysis. After screening titles and abstracts, 21 articles were selected. Additionally, two articles from citations used in included articles and seven reports from government websites of Bangladesh met all eligibility criteria. Overall, 30 studies (20 peer-reviewed articles and 10 grey articles) were included in the scoping review. Figure 1 demonstrates the Preferred Reporting Items for Systematic and Meta-Analyses (PRISMA) flowchart of included articles as detailed above. Additionally, a full list of included documents with summarized findings is provided in Table 3 of the supplementary document.

**Table 3.** Summary of included articles

Reference	Country/setting	Type of article/methodology	Summary of findings
(Mihigo *et al.*, 2015)	Angola Benin Cameroon Comoros Congo Republic Côte d’Ivoire Eswatini Ghana Lesotho Nigeria Zambia Zimbabwe	Programme Activity Report; WHO regional office collected reports from 43 countries in 2013 and 2014. Countries reported on their integrated public health interventions as a part of AVW and also on the successes of their predetermined vaccination goals for AVW	· In 2014 more countries implemented multiple interventions compared with 2013. · Countries increased the number of other interventions they were offering. · Main focus was on creating awareness about immunization through different events. · Some of the interventions that were integrated included vit A administration, growth monitoring, deworming, catch-up vaccination, introducing new vaccines, distributing long-lasting ITNs, distributing WASH kits.
(Doherty *et al.*, 2010)	Ghana India Indonesia Senegal Tanzania Zambia	Systematic review of peer reviewed articles and grey literature between 1970 and 2005 using 48 keywords. Recommendations from field experts and internet searches were used to find grey literature.	· Three types of integration were identified: linked referral, integrated routine delivery and integrated campaign delivery. · The interventions reviewed were: offering family planning services to mothers and giving anti-malarial medication to infants during RI, distributing vitamin A or deworming tablets during campaigns or RI, distributing ITNs through immunization campaigns. · Some countries implemented more than one intervention at a time. · Integration was the most successful and could increase coverage when the immunization program that it was linked to was already robust.
(Molina-Aguilera *et al.*, 2012)	Honduras	Mixed-Method; Reviewing routine vaccination and national vaccination week reports between 1991 and 2009 and interviewing the health secretary and EPI personnel to identify integration activities.	· The identified integration activities can be classified into two categories, health promotion and disease prevention or response to public health emergencies. · Vit A was the first integrated campaign and is assumed to be successful. · Retinoblastoma detection campaigns effectively increased the number of eye tumours that were detected early. · Some campaigns that were dependent on donations were eventually discontinued due to financial restrictions.
(Gorstein *et al.*, 2003)	India	Quantitative Panel; Three rounds of data collection within a 6 month period. Baseline data was collected before the national immunization day. After that, data was collected again at two weeks and sixteen weeks after the national immunization day.	· Bitot’s spots were found in less than half of the children identified during the baseline after four weeks. · Acute respiratory disease and diarrhoeal disease also showed some improvements at 4 weeks and 16 weeks. · This integration campaign increased coverage to 97% in 2000. Overall, short term high dose vit A has a significant effect on those children who have severe deficiencies but the effects last up to 90 days.
(Ching *et al.*, 2000)	Philippines Vietnam	Impact Analysis; Calculation on retrospective data collected by WHO between 1998 and 1999 on immunization integrated with Vit A campaigns	· The cost of integrating vit A into immunization campaigns is very small and vit A reduces child mortality. · The number of countries/immunization campaigns that have incorporated Vit A has doubled since 1997.
(Doherty *et al.*, 2010)	Tanzania Zambia Zimbabwe	Mixed-Method; Programme evaluation in six countries in sub-Saharan Africa using both quantitative (review of routine child health indicators) and qualitative (KII) methods	· CHDs have raised the profile of child survival at different levels from central government to the community in all six countries. · The approach has increased the coverage of vitamin A supplementation and immunizations, especially in previously poorly performing countries. · Similar improvements have not occurred in non-CHD interventions, most notably exclusive breastfeeding. · There were examples of duplication, especially in the capturing and use of health information. · There was widespread evidence that PHC staff were being diverted from their usual PHC functions, and managers reported being distracted by the time required for the planning and execution of CHDs. · There were examples of where the routine PHC system is becoming distorted through, for example, the payment of health worker incentives during CHD activities only
(Vince *et al.*, 2014)	Papua New Guinea	Case Study; no methodology specified	· Integrating interventions during SIA helps maintain polio free status and reduce measles mortality when RI coverage is low. · Integrating multiple interventions is the most cost effective when the target population is small.
(Grabowsky *et al.*, 2005)	Zambia	Quantitative, Post-only; During a national measles vaccination campaign, children in four rural districts were given a free ITN when they received their measles vaccination. About 1700 HHs were asked whether they received vaccination and an ITN during a measles campaign, as well as questions on assets (e.g. type roofing material or bicycle ownership) to assess HH wealth	· ITN coverage among children rose from 16.7% to 81.1% in rural areas and from 50.7% to 76.2% in urban areas. · The operational cost per ITN delivered was $0.35 in the rural area with direct distribution and $1.89 in the urban areas with voucher distribution (not including operational cost of vaccination program)
(Johri *et al.*, 2013)	India	Quantitative Cross-Section; Officers from immunization programs in 14 states were interviewed with a structured questionnaire.	· Positive responses regarding integration of health interventions to measles SIA. · Suggested interventions included vit A supplementation, child and maternal nutrition, ORS saline, deworming and supplementation for pregnant women. · Respondents identified the key focus areas as safety, increased outreach to underserved children and interventions selected by disease burden
(Jacobs *et al.*, 2012)	Laos	Qualitative; Study sites were purposively selected to capture a diverse picture of conditions and experiences with outreach services. Conducted 58 IDIs with district representatives and provincial health offices, health centre staff and village health volunteers. Vaccination outreach sessions by health centre staff were observed in eight villages and 12 FGDs were held with 120 mothers on their perceptions of these sessions.	· The regularity and frequency of outreach sessions and the number of integrated vaccination/MCH services varied widely between sites. · Availability of external financial and technical support was the major determinant of optimal delivery of integrated services. · To enable concurrent delivery of a range of MCH services during vaccination outreach, the number of these services should gradually be increased in tandem with additional financial and technical support.
(Watson-Jones *et al.*, 2016)	Tanzania	Desk review of 39 policy documents; preceded by a stakeholder meeting with 38 policy makers and partners. 18 KIIs with health and education policy makers and district officials were conducted to further explore perceptions of current programs, priorities and AHI that might be suitable for integration with HPV vaccination	· 14 school health interventions (SHI) or AHI are currently being implemented by the Gov. of Tanzania. Most are delivered as vertical programmes. · Deworming and educational sessions including reproductive health education were the most frequently mentioned interventions that respondents considered suitable for integrated delivery with HPV vaccine · Given programme constraints, limited experience with integrated delivery and concern about real or perceived side-effects being attributed to the vaccine, it will be very important to pilot-test integration of AHI/SHI with HPV vaccination. · Selected interventions will need to be simple and quick to deliver since health workers are likely to face significant logistic and time constraints during vaccination visits
(Agtini *et al.*, 2006)	Indonesia	Randomized Control Trial; Report of vaccine campaign. 18 schools from two states of North Jakarta were randomly selected to provide Vi polysaccharide vaccine through a vaccine campaign. A mop up campaign was also conducted. Information dissemination activities were carried out three months before the intervention. All vaccinations were recorded during the intervention.	· 91% coverage was achieved among those who were eligible. · The campaign was found to be logistically feasible and safe. · Using the resources of an existing, successful immunization campaign was found to be effective. · The pre-planned mop up campaigns increased vaccine coverage. · Mop up campaigns were not used during BIAS but implemented for this specific program. · Although mop up campaigns required additional time and resources, they increased coverage.
(Habib *et al.*, 2017)	Pakistan	Community-based three-arm cluster randomized trial. Clusters received either routine polio programme activities (control, arm A), additional interventions with community outreach and mobilization using an enhanced communication package and provision of short-term preventive MNCH services and RI (health camps), including OPV (arm B), or all interventions of arm B with additional provision of IPV delivered at the maternal and child health camps (arm C)	· Results indicated widespread acceptance, with more than two-thirds of target children and families in these districts accessing short-term fixed camps during the 4 days of the campaigns. · The coverage of OPV increased 8.5% overall, uptake of inactivated polio vaccine administered alongside OPV was high, and the number of children who were not immunized was reduced overall
(Mwingira *et al.*, 2016)	Tanzania	Programme Activity Report; Analysis of integration of Immunization program scheduled to deliver a supplementary MR vaccination campaign and NTD MDA campaign scheduled to distribute ivermectin and albendazole. The MR campaign targeted 27 of the country’s implementation units, with 16 of the areas also targeted by the NTD campaign.	· Routinely reported program coverage of the MDA program increased from 86% in 2013 to 93% in 2014 following additional mop-up activities. · MR vaccination coverage remained high with 97% coverage in 2014 relative to the previous campaign in 2011 that achieved 96% coverage · The joint campaign increased coverage relative to previous vertical campaigns by reducing the amount of time community members expended on community-based health-care activities and increasing the perceived incentives associated with a single health-care event. · The MDA program benefited from the high demand of the popular immunization campaign · Program costs may have increased with the coordinated campaign. This observed increase in budgeted financial costs is probably due to a number of factors: including greater coverage & services of individual programs on top of unique start-up costs associated with launching a newly integrated program. · It was not possible to compare the disaggregated expenditures of the campaigns given that itemized activity and input costing exercises were not conducted
(Ryman *et al.*, 2012a)	Cameroon Kenya	Mixed-Method; Comparison of two intervention strategies. Interventions were assigned randomly at cluster level (two regions with 9 clinics in each) with a pre-post-intervention survey for assessment of hygiene indicators and vaccination coverage; and IDIs and FGD to assess acceptability.	· 78% of caregivers received a hygiene kit as opposed to 22% who had not been vaccinated, thus never exposed to the intervention; with more distributed at the nurse clinics (56%) than at the SWAP clinics (44%). · Increase in observed and reported practice of using hygiene intervention (WaterGuard) was high in rural areas but not in urban sites, where baseline was already high. · Change in other hygiene knowledge and practice indicators were observed with similar patterns across various distribution strategies. · “Implementation coverage” and up-to-date coverage significantly increased in urban nurse sites, with no change in either nurse or SWAP rural sites · The integrated approach was well accepted in both nurse and SWAP sites by nurses, mothers and SWAP members, with no major criticisms of either of the strategies. · The intervention motivated mothers/caregivers to have their children vaccinated who had not yet done so or would not otherwise.
(Wallace *et al.*, 2017)	Nepal	Quantitative Pre-/Post-; 100 randomly selected health facilities were surveyed before and after the SIA and evaluated for: vaccine safety, RI planning, RI service delivery, vaccine supply chain and RI data recording practices. Data collectors interviewed the person who had given the most vaccines at each health facility using a structured questionnaire. Pair-matched analytical methods were used to determine whether statistically significant changes occurred in the selected RI process measures due to SIA integration	· Nepal had 90% RI coverage for these vaccines to begin with. · The improvements with integrating SIA might be helpful to maintain this high coverage. · SIA showed improvements in some components of RI such as slight improvements to the vaccine cold chain and to certain areas of vaccine knowledge. · There were some significant decreases in some aspects of immunization knowledge after SIA.
(Velleman *et al.*, 2013)	Nepal	Semi-structured Qualitative; FGD and KII during the national polio week. Questions were related to whether hygiene interventions should be implemented and about how they should be integrated	· All respondents were positive regarding integration. · Most respondents believed that integrating during RI would be the best. This would allow hygiene interventions to be carried out more frequently and not be rushed. · Most respondents believed that implementation should be done by FCHV workers whom the community already trusts and not through other groups/posters/flyers.
(Suwantika *et al.*, 2020)	Indonesia	Quantitative Cohort; Age-structured decision tree model was developed to assess cost-effectiveness. Comparison of three vaccination strategies: (1) focusing on vaccination only, (2) combining vaccination and a Wolbachia program and (3) combining vaccination and health education. All scenarios were compared with a no-intervention strategy	· The combination of vaccination and a Wolbachia program would reduce dengue fever (DF), dengue haemorrhagic fever (DHF) and dengue shock syndrome (DSS) by 292 488; 230 541; and 672 cases, respectively. · It would also save treatment cost at $24.3 million and $14.6 million from the healthcare and payer perspectives, respectively. · The incremental cost-effectiveness ratios (ICERs) from the healthcare perspective were estimated to be $4460 per quality-adjusted life year (QALY) gained for the scenario. · The vaccination combined with a Wolbachia program was confirmed to be the most cost-effective intervention.
(Deardorff *et al.*, 2018)	Bhutan Bolivia Cambodia Cameroon Egypt Ghana Haiti Honduras India Kenya Lesotho Nepal Nigeria Pakistan Philippines Tanzania Uzbekistan Zambia Zimbabwe	Systematic review of CADdirect, MBase and PubMed on community-based health campaigns, which reported two or more treatment coverage estimates	· Increasing health facility distributor incentive by hiring them permanently · Community directed treatment distribution using kinship groups of relatives in the community, (in places where relatives live near each other) · Enhanced information education and communication (IEC) activities · School based service delivery for children and NGO delivery instead of MOH delivery. · Community based treatment delivery had the largest impact and highest coverage when combined with another strategy such as kinship groups. NGO management also showed the highest absolute coverage
(Ryman *et al.*, 2012b)	Cameroon Kenya	Semi-structured qualitative KIIs with health workers and FGDs with the community in four countries. The questions were on health services other than immunization, methods of integration, satisfaction with integration and challenges and suggestions for improvement. The interview sites were selected purposively.	· Response was positive in Kenya among both groups of respondents. · Community members and health workers agreed that the integration increased immunization attendance. · In Cameroon respondents felt that integration saved time and protected their privacy. · Mothers could access different health facilities during a single visit and HIV patients felt that seeing one person for everything protected their privacy. · Health workers mostly showed concerns about inadequate staffing and felt that incentives would increase their productivity. · Community members in both countries felt that the success of integration is mostly owed to the education component of the intervention.
(Biellik *et al.*, 2009)	India Vietnam	Descriptive Qualitative study that synthesized the health system components from formative research implemented in four developing countries conducted from 2006 to 2008	· Consensus that the EPI represents potentially the most effective mechanism for delivering the HPV vaccines, in view of its established national reputation, infrastructure and well-trained HR · Full integration of the HPV vaccines into routine vaccine service delivery through fixed health facilities and mobile teams serving outreach sites was generally not considered viable by health workers and technical staff at the district level in India, Peru and Uganda
(Akhter *et al.*, 2008)	Bangladesh	Quantitative Cross-Sectional	· The National Vitamin A Plus Campaign (NVAC) started in 2003 with an objective to sustain the less than 1% prevalence of night blindness among children aged less than 5 years · An integrated approach was used to deliver multiple interventions that included deworming, oral polio vaccines and awareness campaigns on breastfeeding and nutrition
(World Health Organization, 2018)	Cambodia Cameroon Congo Republic Ghana Honduras India Kenya Laos Nepal Nigeria Philippines Tanzania Vietnam Zambia Zimbabwe	*Grey Literature* Resource Guide, Report	· This resource guide brings together a range of relevant resources, summarizes the knowledge and provides guidance on the integration of immunization with other health interventions, health policies or activities to strengthen health systems · In Kenya distribution of hygiene kits increased vaccine coverage in urban settings, but were not sufficient to overcome access barriers in rural areas · In Zimbabwe decentralize HIV follow-up counselling services was integrate with immunization clinics
(Directorate General of Health Services, 2013a)	Bangladesh	*Grey Literature,* Campaign guide	Provides detailed guidelines on operational procedures of Measles and Rubella campaign integration to national immunization day campaign Content details on campaign preparation i.e. microplanning, campaign management, reporting etc.
(Directorate General of Health Services, 2013b)	Bangladesh	*Grey Literature,* Health Bulletin	Vitamin A and deworming added to the National Immunization campaign day.
(Directorate General of Health Services, 2014)	Bangladesh	*Grey Literature,* Health Bulletin	Coverage of Oral Polio Vaccine, Measles and Rubella immunization, Vitamin A and deworming reported to increase due to integration to National Immunization Campaign day
(Directorate General of Health Services, 2015)	Bangladesh	*Grey Literature,* Health Bulletin	Similar reporting as previous year
(Directorate General of Health Services, 2016)	Bangladesh	*Grey Literature,* Health Bulletin	Similar reporting as previous years
(Directorate General of Health Services, 2017)	Bangladesh	*Grey Literature,* Health Bulletin	Similar reporting as previous years
(Directorate General of Health Services, 2018)	Bangladesh	*Grey Literature,* Health Bulletin	Similar reporting as previous years

### Characteristics of the included documents

The studies reviewed used a variety of methodologies: quantitative (*n* = 6), qualitative (*n* = 4), mixed-method (*n* = 3), systematic reviews (*n* = 3), non-experimental case studies (*n* = 3), randomized control/cohort trials (*n* = 2), one impact analysis and one program campaign guide.

The studies also covered a wide range of LMICs; however, there were several notable gaps, including most American, Central Asian and Pacific Island LMICs. Additionally, although the study by Mihigo *et al.* (2015) discussed African Vaccination Week in all African Region Member States, only the countries that participated in IHC activities are included in this review. Table 4 lays out the list of integration by source.

**Table 4.** List of integration by source

Source		Integrated services	
Reference	Country	Immunization services	Other health services
(World Health Organization, 2018)	Cambodia, Cameroon, Congo Republic, Ghana, Honduras, India, Kenya, Laos, Nepal, Nigeria, Philippines, Tanzania, Vietnam, Zambia, Zimbabwe	Routine immunization	Hygiene kits HIV follow-up counselling
(Mihigo *et al.*, 2015)	Angola, Benin, Cameroon, Comoros, Congo Republic, Côte d’Ivoire, Eswatini, Ghana, Lesotho, Nigeria, Zambia, Zimbabwe	Each country implemented its own integration of services Polio campaign Catch-up vaccination activities New vaccine introduction: Rotavirus Pneumococcal vaccine (PCV) HPV Measles (2nd dose)	Each country implemented its own integration of services Communication activities (e.g. advocacy, social mobilization, etc.) Vitamin A administration Antihelminthics distribution Malnutrition screening ITN distribution
(Doherty *et al.*, 2010)	Ghana, India, Indonesia, Senegal, Tanzania, Zambia	Child immunization	Family planning services Antimalarial distribution ITN distribution Vitamin A supplementation Antihelminthics distribution
(Molina-Aguilera *et al.*, 2012)	Honduras	National vaccination week Door-to-door immunization	Vitamin A supplementation Folic acid supplementation for women of childbearing age Retinoblastoma detection in children under 5 Promoting breastfeeding, Dengue and cholera prevention activities
(Gorstein *et al.*, 2003)	India	Polio vaccination	Vitamin A supplementation
(Ching *et al.*, 2000)	Philippines Vietnam	Various (national) immunization campaigns	Vitamin A supplementation
(Doherty *et al.*, 2010)	Tanzania Zambia Zimbabwe	Measles vaccination	Vitamin A supplementation ITN distribution during CHD
(Vince *et al.*, 2014)	Papua New Guinea	Measles and Polio vaccination Tetanus toxoid immunization for women of child-bearing age Routine childhood vaccination	Vitamin A supplementation Antihelminthics distribution
(Grabowsky *et al.*, 2005)	Zambia	Measles vaccination	ITN distribution
(Johri *et al.*, 2013)	India	Measles SIA	Vitamin A supplementation Child and maternal nutrition ORS saline distribution Antihelminthics distribution Supplementation for pregnant women
(Jacobs *et al.*, 2012)	Laos	Child immunization	Maternal and child health services
(Watson-Jones *et al.*, 2016)	Tanzania	HPV vaccination	Adolescent health interventions
(Agtini *et al.*, 2006)	Indonesia	School-based immunization program Introducing of vi polysaccharide typhoid vaccine	
(Habib *et al.*, 2017)	Pakistan	OPV Inactive polio vaccine	Maternal and child health services
(Mwingira *et al.*, 2016)	Tanzania	MR vaccination	Antihelminthics distribution
(Ryman *et al.*, 2012a)	Cameroon Kenya	Routine vaccination (incl. BCG, OPV and penta)	Hygiene interventions
(Wallace *et al.*, 2017)	Nepal	Routine immunization MMR SIA	
(Velleman *et al.*, 2013)	Nepal	Polio vaccination	Hygiene interventions
(Suwantika *et al.*, 2020)	Indonesia	Dengue vaccination	Wolbachia program (for dengue prevention)
(Deardorff *et al.*, 2018)	Bhutan, Bolivia, Cambodia, Cameroon, Egypt, Ghana, Haiti, Honduras, India, Kenya, Lesotho, Nepal, Nigeria, Pakistan, Philippines, Tanzania, Uzbekistan, Zambia, Zimbabwe	Measles vaccination	Vitamin A supplementation
(Ryman *et al.*, 2012b)	Cameroon, Kenya	Routine vaccination services	Antenatal consultation Antihelminthics distribution Health education HIV/AIDS prevention and treatment Malaria prevention (e.g. ITNs, etc.) Tuberculosis treatment Vitamin A supplement distribution
(Biellik *et al.*, 2009)	India, Vietnam	Various (national) immunization programs Introduction of HPV vaccines	
(Akhter *et al.*, 2008)	Bangladesh	OPV	Vitamin A supplementation Deworming Breastfeeding awareness and nutrition

### Summary of findings on IHCs in immunization in LMICs

#### Common strategies for effective integration

Across the board, the articles reviewed showed that the integration of campaigns has a positive impact on the coverage and/or effectiveness of vaccination campaigns. Strategic implementation paved the way for better coverage of services. Some of these strategies included: using integrated resources efficiently in remote locations; using national immunization days (NIDs) to maximize impact; targeting specific age groups by selecting intervention sites that are frequented by that age group; and building community ownership over the integrated program. Here, we examine each of these strategies in more detail.

#### Improved coverage in remote areas

Integration as a means of increasing coverage in remote areas was the most thoroughly explored sub-topic in the review, discussed in five of the documents (Doherty *et al.*, 2010; Vince *et al.*, 2014; Grabowsky *et al.*, 2005; Johri *et al.*, 2013; Jacobs *et al.*, 2012; Watson-Jones *et al.*, 2016; Agtini *et al.*, 2006; Biellik *et al.*, 2009). Three of these studies reported the success of integrating child immunization with maternal and child health (MNCH) services in remote regions (Vince *et al.*, 2014; Johri *et al.*, 2013; Jacobs *et al.*, 2012). For example, the remoteness of Papua New Guinea makes it challenging to implement routine immunization. The coverage of supplementary immunization activities involving polio and measles immunization increased when integrated with a package of MNCH services such as tetanus toxoid immunization, routine childhood vaccination, vitamin A supplementation and deworming for children (Vince *et al.*, 2014). In Laos, the burden of maternal and child mortality was high due to low coverage of MNCH services in difficult-to-access regions. To increase the coverage of MNCH services, integration was done using the more established platform of immunization outreach programs This campaign was positively received by women since it lifted the burden of having to travel to the city to access antenatal care services, while simultaneously being ‘good for the babies’ (Jacobs *et al.*, 2012).

#### Nationwide immunization campaigns

Several articles in the review focused on nationwide immunization days, weeks, or months (World Health Organization, 2013; Mihigo *et al.*, 2015; Doherty *et al.*, 2010; Grabowsky *et al.*, 2005; Molina-Aguilera *et al.*, 2012). Two multi-country studies in our review reported that the integration of services during such intermittent national campaigns increased coverage (Doherty *et al.*, 2010; Mihigo *et al.*, 2015). One of the studies explored the integration of a variety of child survival interventions, introduced in sub-Saharan Africa during Child Health Day (CHD). The goal of CHD was to respond to the declining coverage of interventions that would help prevent under-five child mortality. As a result, the integration of vitamin A supplementation, immunization (notably for measles) and ITN distribution during CHD increased the coverage of these services in previously poorly performing sub-Saharan African countries (Doherty *et al.*, 2010). Similarly, a review of WHO regional office reports on country-specific integrated health interventions implemented during African Vaccination Week, between 2013 and 2014 in 43 African nations, also found that integration increased overall vaccine coverage (Mihigo *et al.*, 2015). The interventions included vitamin A supplementation, child growth monitoring, catch-up vaccination, introduction of new vaccines, and distribution of water, sanitation and hygiene (WASH) kits and ITNs. Furthermore, the participating countries increased the number of integrated health services during the same period, which could be an indication that integration was beneficial in achieving healthcare targets (Mihigo *et al.*, 2015). Consistent with these findings, the provision of ITNs during the national measles immunization campaign in Zambia increased ITN ownership in the study areas. The increase in ITN coverage was significant in both rural and urban areas, with an increase from 16.7% to 81.1% in rural areas and 50.7% to 76.2% in urban areas, based on a post-campaign survey (Vince *et al.*, 2014).

#### School-based implementation when the target population is children/ adolescents

Implementing a school-based approach when the target population is under 18 has been shown to have positive effects on the coverage and cost-effectiveness of integrated programs (Watson-Jones *et al.*, 2016; Agtini *et al.*, 2006; Biellik *et al.*, 2009). Adolescent girls generally have fewer contacts with healthcare providers and school-based human papillomavirus (HPV) immunization campaigns achieved good coverage within this target group in Tanzania, where they were coupled with Adolescent Health Interventions (a package of 14 curriculum and non-curriculum-based interventions targeting sexual and reproductive health, sanitation and hygiene education, immunizations, neglected tropical diseases (NTDs), school health assessments and nutrition). Most stakeholders and health service providers interviewed agreed that approaching adolescent girls in schools was the most cost-efficient way of providing HPV vaccines (Watson-Jones *et al.*, 2016). School-based integration is also helpful for introducing new vaccines that target school-aged children. In Indonesia, a successful school-based BIAS program (a large-scale, month-long immunization program for school-going children) was used to introduce a new Vi polysaccharide typhoid vaccine, which resulted in 91% coverage (Agtini *et al.*, 2006). Since very few studies in our review assessed school-based integration campaigns, there is potential for future research on how integration may further increase the coverage and cost-effectiveness of these programs.

#### The most frequented integration: with vitamin a supplementation

In the studies reviewed, vitamin A supplementation was the most frequently discussed health intervention to be integrated with vaccination programs (Mihigo *et al.*, 2015; Doherty *et al.*, 2010; Johri *et al.*, 2013; Bhatnagar and Gittelman, 2020; Vince *et al.*, 2014; Grabowsky *et al.*, 2005; Jacobs *et al.*, 2012; Watson-Jones *et al.*, 2016; Agtini *et al.*, 2006; Biellik *et al.*, 2009; Molina-Aguilera *et al.*, 2012; Suwantika *et al.*, 2020; Deardorff *et al.*, 2018; Ryman *et al.*, 2012b; Ching *et al.*, 2000; Directorate General of Health Services, 2013a; 2013b; 2014; 2015; 2016; 2017; 2018). In a review of studies that exclusively integrated vitamin A supplementation with immunization, increased coverage of vitamin A was observed in all cases. Additionally, in studies where it was measured, immunization coverage also increased (Molina-Aguilera *et al.*, 2012; Ching *et al.*, 2000; Wallace *et al.*, 2009; Gorstein *et al.*, 2003). Vitamin A was shown to reduce child mortality at minimal cost when it was integrated into child immunization programs, in a study done in Vietnam (Habib *et al.*, 2017). However, another study indicated that high doses only have a significant impact on children with severe deficiencies and even then, for only up to 90 days (Gorstein *et al.*, 2003). Therefore, our review suggests that although vitamin A supplementation must be maintained intermittently, it is still beneficial to integrate it into other health campaigns.

#### Community involvement for successful implementation

A handful of documents in this review touched upon community involvement in IHC implementation (Biellik *et al.*, 2009; Habib *et al.*, 2017; Mwingira *et al.*, 2016; Ryman *et al.*, 2012a). One of the mechanisms behind increased coverage is that integration allows healthcare workers to develop stronger trust and rapport with the community when they offer multiple services, including services that already have high acceptance within the community. Services that have low acceptance and trust in the community, when coupled with services that are already widely accepted, can increase coverage for the former. For example, when MNCH services were integrated with the less accepted child polio vaccination in conflict-affected and polio-endemic areas of Pakistan, the coverage and uptake of the OPV went up by 8.5% (Habib *et al.*, 2017). In contrast, immunization is positively accepted in Tanzania due to years of Gavi, the vaccine alliance’s advocacy. Linking a mass drug administration (MDA) campaign for parasitic diseases to Tanzania’s popular measles-rubella immunization campaign increased coverage and trust for the MDA campaign (Mwingira *et al.*, 2016). Increasing cultural acceptance of health services is also closely related to maintaining the privacy of patients. In Cameroon, women reported having their privacy protected through the integrated co-delivery of the human immunodeficiency virus (HIV) and other maternal health services with anthelmintic and vitamin A supplementation for children. This allowed HIV-positive mothers to see the same health professional for their illness as well as their child’s health needs, keeping their health condition confidential (Ryman *et al.*, 2012b).

#### Integration of complementary health campaigns and routine services

The concept of complementary health campaigns was explored by some documents in our review (Doherty *et al.*, 2010; Suwantika *et al.*, 2020; Wallace *et al.*, 2017; Velleman *et al.*, 2013). As discussed below, complementary campaigns generally have a shared outcome, whether it is vaccine coverage or tackling specific issues such as diarrhoeal diseases or dengue. Integrating health campaigns that complement each other is effective for increasing the impact of each campaign. In Nepal, the coverage of measles, mumps and rubella (MMR) vaccines is 90%. An intervention of supplementary immunization activities for MMR vaccination was designed to strengthen certain aspects of RI and maintain this state of high coverage. As a result, this integration improved knowledge of some aspects of RI, such as cold chain management and vaccine knowledge (Wallace *et al.*, 2017). Another study in Nepal was carried out preceding the introduction of a rotavirus vaccine to determine the feasibility of integrating hygiene interventions with rotavirus immunization. This intervention aimed to avoid the possible misconception in the community that rotavirus vaccines are ‘diarrhea vaccines that can prevent all forms of diarrhea’ and emphasize the importance of combined approaches of WASH and immunization to prevent diarrhoeal diseases. Interviewed health providers agreed that this integration method will be successful in preventing all aspects of diarrhoeal diseases simultaneously (Velleman *et al.*, 2013). Another example of complementary integration is dengue prevention with combined dengue immunization and a Wolbachia program (Wolbachia are parasitic bacteria that only infect insects and outcompete the dengue virus within the mosquito’s body). A case study on its feasibility carried out in Indonesia found that integration is necessary since no single intervention is sufficient to combat dengue (Suwantika *et al.*, 2020).

#### Implementation of integrated health campaigns: facilitators

Just as many of the included documents featured common implementation strategies to improve coverage, the documents also featured common facilitators for effective integration. These strategies included: developing community ownership, allocation of adequate time/resources for planning, striving for cost-effectiveness and utilization of pre-existing infrastructure. These points are discussed in detail below.

#### Community ownership & closing the gap between services and community

The most common facilitator of successful implementation mentioned by the included documents was the establishment of community ownership (Mihigo *et al.*, 2015; Jacobs *et al.*, 2012; Deardorff *et al.*, 2018; Ryman *et al.*, 2012a; Habib *et al.*, 2017; Akhter *et al.*, 2008). A systematic review of African LMICs found that community involvement in decision-making and service delivery was the reason behind the increased coverage of a variety of programs (Deardorff *et al.*, 2018). In the case of integrating new or culturally sensitive health services, one effective strategy was to pair them up with an already popular health program. In this case, the high demand and community acceptance of the old program acted as an incentive for embracing the new program. For example, Family Planning or HIV services integrated with other programs provided patient confidentiality, which in turn increased community acceptance of the IHC. Patient confidentiality was achieved by allowing individuals to avail of stigmatized services under the guise of availing of the more popular service.

There are some effective strategies for responding effectively to community needs. In hard-to-reach communities, it is important to establish that the integrated program is closing the gap between healthcare seekers and providers (Jacobs *et al.*, 2012; Deardorff *et al.*, 2018). Service receivers are more willing to meet providers halfway by delaying other responsibilities, knowing that this is preferable to having to make the trek to the nearest health provider themselves (Jacobs *et al.*, 2012). Afterwards, the coverage of these programs remains high, even after the initial intervention is complete (Deardorff *et al.*, 2018). Across contexts, the recruitment of community and religious leaders is valuable for building community acceptance.^32^

#### Coordination, planning & resource management

Along with the community element, the other most common facilitator of effective integration was ensuring adequate planning and resource management both before and throughout the IHC process (Mihigo *et al.*, 2015; Molina-Aguilera *et al.*, 2012; Ryman *et al.*, 2012b; Mwingira *et al.*, 2016; Ryman *et al.*, 2012a; Akhter *et al.*, 2008). The three most important elements within this category were planning before the launch of the program (Mihigo *et al.*, 2015; Molina-Aguilera *et al.*, 2012; Ryman *et al.*, 2012a; Ching *et al.*, 2000; Wallace *et al.*, 2009; Gorstein *et al.*, 2003; Habib *et al.*, 2017; Mwingira *et al.*, 2016; Ryman *et al.*, 2012b; Wallace *et al.*, 2017; Velleman *et al.*, 2013; Akhter *et al.*, 2008), effective training of staff (Molina-Aguilera *et al.*, 2012; Ryman *et al.*, 2012a) and effective monitoring and evaluation efforts throughout the run of the program (Molina-Aguilera *et al.*, 2012; Wallace *et al.*, 2009; Mwingira *et al.*, 2016). About prior planning, it was imperative that an accurate accounting of the required intervention materials be maintained (Mihigo *et al.*, 2015) and that there be transparency between the integrators (Molina-Aguilera *et al.*, 2012). It should also be noted that these efforts are simplified when the integrated programs are compatible. Compatible programs share at least one or more of the following aspects: target populations, logistical needs, worker training requirements, stakeholders and supply chain requirements (Agtini *et al.*, 2006). This allows for the deployment of already trained staff (Agtini *et al.*, 2006) and the elimination of redundancies in general (Mwingira *et al.*, 2016).

#### Cost-effectiveness

Despite prior assumptions, the cost-effectiveness of the IHC strategy was a less frequently cited facilitator to IHC implementation, compared with those previously mentioned (Grabowsky *et al.*, 2005; Watson-Jones *et al.*, 2016; Suwantika *et al.*, 2020; Ching *et al.*, 2000). One case study of Papua New Guinea’s supplementary measles immunization activities specified that integration is the most cost-effective means of delivering several interventions to a small target population (Ching *et al.*, 2000). This sentiment was echoed by another study looking to deliver HPV vaccinations to school-aged girls in Tanzania (Watson-Jones *et al.*, 2016). Integration of ITN distribution with an existing national measles immunization campaign was also found to be cost-effective in a study done in Zambia. Integration reduced the operational cost to a reasonable $0.35 per ITN in rural areas and $1.89 per ITN in urban areas. This addition was found to be the most efficient and cost-effective method of ITN delivery in remote regions of Zambia (Grabowsky *et al.*, 2005).

#### Pre-existing infrastructure for service delivery

Pre-existing infrastructure such as healthcare programs or various outreach programs were identified as a key factor leading to the successful implementation of integrated healthcare in several LMICs (Jacobs *et al.*, 2012; Biellik *et al.*, 2009; Suwantika *et al.*, 2020; Mwingira *et al.*, 2016). When the target population is diverse and involves different age groups, the best method of implementation was using a mix of health posts such as schools, health facilities and community vaccination posts for reaching the entire target population (Mwingira *et al.*, 2016). The use of schools is especially efficient when considering programs that target children and adolescents (Biellik *et al.*, 2009). Additionally, knowing the size of the target population can greatly improve the effectiveness of programs intended to cover hard-to-reach areas. A well-functioning vital registration system can save both time and money (Jacobs *et al.*, 2012). Conversely, the lack of pre-existing infrastructure was also claimed as a potential facilitator in at least one study examining the feasibility of integrating multiple dengue programs into a national campaign. Here, the lack of existing infrastructure would have allowed for the planning of an efficient program from the ground up (Suwantika *et al.*, 2020).

#### Implementation of integrated health campaigns: barriers

The documents also featured common barriers to effective integration. In general, they can be divided between issues related to the costs of integration and issues related to the management of human resources.

#### The costs of integration

Integration can carry costs in the short term. Expansion of healthcare programs even through the cost-effective process of integration may not always be feasible for resource-strapped LMICs (Suwantika *et al.*, 2020; Mwingira *et al.*, 2016; Ryman *et al.*, 2012a; Wallace *et al.*, 2017; Velleman *et al.*, 2013). For example, in 2014, the Government of Tanzania integrated its measles and rubella immunization campaign with its less popular MDA campaign of several NTDs. Surprisingly, the program costs of the IHC were 7.19 million USD, while the combined program costs of the two discrete programs the previous year were 6.04 million USD. As no itemized expenditures were available, it is difficult to determine how much of this increase is due to increased coverage and services provided between the two years, and how much is due to various unique start-up costs from the integration process (Mwingira *et al.*, 2016). Another study examined the feasibility of integrating a dengue vaccination program along with other dengue prevention methods into Indonesia’s existing immunization program and concluded that nationwide implementation may not be possible. Specifically, a budget of approximately ∼90% of the RI budget would be required, which is already 6% of the national healthcare budget (Suwantika *et al.*, 2020). Significant political lobbying or outside funding would be required to procure the necessary funds for the hypothetical dengue programs discussed. These two studies show that program organizers must be aware of the inherent costs of the integration process and ultimately a certain amount of catalyst funding is required to reap the benefits of a highly efficient healthcare program down the line.

#### Human resource challenges

Nearly every paper that examined an ongoing or completed IHC or the feasibility of conducting an IHC cited HR as a major challenge or barrier (Doherty *et al.*, 2010; Grabowsky *et al.*, 2005; Jacobs *et al.*, 2012; Watson-Jones *et al.*, 2016; Agtini *et al.*, 2006; Biellik *et al.*, 2009; Wallace *et al.*, 2009; Mwingira *et al.*, 2016). Several studies mentioned that integration would mean staff on the ground would need to carry and manage more equipment than normal. One review examining the feasibility of an IHC cited the logistics of supporting the overburdened staff as a major issue (Watson-Jones *et al.*, 2016). Similarly, another study, which involved staff travelling to hard-to-reach areas, found that the increase in necessary equipment led to greater wear, tear and loss of said equipment than was expected when the integrated programs were run separately (Jacobs *et al.*, 2012).

Another common issue for staff is the additional workload. One review discussed overburdened staff as a major challenge to integration. This issue was most apparent when staff was not properly trained for each of the integrated tasks (Wallace *et al.*, 2009). Another study that examined the integration of HPV vaccines in multiple countries cited the concern that overburdened health workers would not only impact the program’s effectiveness but also community relations (Biellik *et al.*, 2009).

Successful implementation of any integration program requires the training of field staff to deliver multiple products or services simultaneously. In the case of programs that have the same delivery method, workers must be trained separately for practices such as needle safety (Agtini *et al.*, 2006) or communicating with the local community (Jacobs *et al.*, 2012). This training also has additional costs, which must be accounted for in the planning stage (Watson-Jones *et al.*, 2016). Additional HR costs can also be incurred due to the impossibility of fully removing redundancies when combining staff. In the case of one program, which integrated an MDA program and an immunization program, the MDA program utilized the services of volunteers who relied on per diems from the campaign for their livelihoods. These volunteers could not be trained to handle the needles required for vaccinations (as this was taken care of by the healthcare workers already employed for the immunization program), but they could also not be let go, as this would negatively affect the community perception of the program (Mwingira *et al.*, 2016).

#### Recommendations from the included articles

Only one article mentioned a recommended list of actions that have worked in the successful implementation of IHCs in the African LMICs (Molina-Aguilera *et al.*, 2012). The courses suggest coordination between existing programs or departments of the health ministry, joint planning at various health-system levels regarding the programs, supplies and development of technical guidelines, training and supervision of personnel and adaptation of computerized documentation forms and information systems (Molina-Aguilera *et al.*, 2012). A national committee to carry out the promotion of such campaigns and social communication among stakeholders, partners, donors and populations was also recommended for IHCs (Molina-Aguilera *et al.*, 2012).

#### A case of Bangladesh: lessons on integration experiences from grey literature

We examined the work of Bangladesh through grey literature as a peripheral part of the review, as the principal researchers of this study are based in the country and have familiarity with the history of integration in Bangladesh before conducting the review, found that integrating campaigns is a key case in point.

The Bangladesh National Immunization program, introduced in 1995, was used to maintain the country’s polio-free status while simultaneously providing a platform for the integration of other programs, such as vitamin A supplementation (Akhter *et al.*, 2008). A more recent example of IHCs in Bangladesh is the 2013 MR immunization campaign, which was combined with Bangladesh’s NID. The MR campaign shared most of the NID’s resources including micro-planning, HR, volunteers, infrastructure, logistics, community engagement methods, training and advocacy meetings (Directorate General of Health Services, 2013a). Vitamin A supplementation and deworming were also added to this platform of integrated immunization campaigns (Directorate General of Health Services, 2013b). This integration resulted in high coverage of OPV, MR immunization, vitamin A and deworming (Directorate General of Health Services, 2013b; 2014; 2015; 2016; 2017; 2018).

## Discussion

### Summary of findings

In general, this review found that IHCs nearly always improve the coverage and/or effectiveness of the health campaign(s) being examined. This was true for both papers that examined a specific IHC and those that considered a hypothetical program. Successful programs are facilitated by creating community ownership, ensuring comprehensive pre-planning and day-to-day monitoring, improving cost-effectiveness and utilizing pre-existing infrastructure. The two major barriers faced by IHCs are the cost of integration and human resource challenges.

Beyond the identification of facilitators and barriers, several points of commonality were also discovered among the disparate integrated programs IHCs were particularly effective in improving coverage in remote areas. Similarly, IHCs which targeted schools were effective in reaching children, adolescents and their mothers. Generally, IHCs which can involve the community are more likely to last. A common goal for IHCs is to improve the coverage of a novel or stigmatized program. This was most often done through integration with a nationwide immunization campaign, a complementary program, or a more socially-accepted program.

### HR and costs: double-edged swords

Both HR considerations and cost were highlighted in our initial research as chief incentives for LMICs to embrace IHCs (The Task Force for Global Health, 2020). Based on this scoping review, however, improved cost-effectiveness was one of the lesser cited facilitators of effective IHCs (mentioned in four articles) (Grabowsky *et al.*, 2005; Watson-Jones *et al.*, 2016; Suwantika *et al.*, 2020; Ching *et al.*, 2000). Conversely, the prohibitive cost of integration was cited more often as a barrier against its implementation. At least one study found the joint program cost to be greater than the sum of separate programs the prior year, while others mentioned that proposed IHC plans were not feasible without outside funding. Similarly, HR challenges beset nearly every relevant study examined. Issues ranged from overburdening to lack of sufficient training. Nevertheless, a highly trained and motivated staff in the field is essential to the successful implementation of any program. Those programs were able to carefully plan around and utilize their HR and identify them as an important facilitator of successful implementation.

The takeaway here is not that integration is always a burden in terms of cost or HR issues, but rather, that with careful planning and coordination, integration can increase cost-effectiveness and streamline workflows for frontline staff over time.

### Compatibility: broadening target populations

To make the most of increased coverage through IHCs, our review found that the compatibility of the integrated services plays a significant role. A direct finding from a systematic review included states that the compatibility characteristics of different services are similarities in the target population, logistical needs, worker training, stakeholder support, cost and supply chain requirements (Doherty *et al.*, 2010). As such, compatibility can be addressed in many ways such as maximizing demographic overlap of the target populations, integrated services and infrastructural or logistic homogeneity.

Compatible target populations do not need to be solely homogenous. For example, in MNCH services, the target population of mothers and their children, are highly conducive to the integration of various services. Services integrated such as supplements and tablets, basic antenatal care, family planning, health promotion, education, etc. (Wallace *et al.*, 2012), with regular outreach immunization campaigns—which can be compared with IHCs in context immunization—have been found difficult to implement when not having an adequate overlap of target populations (Sobanjo-ter Meulen *et al.*, 2015; Perry *et al.*, 2017). The strategy has also been explored in the areas of HIV services, where HIV screening and testing of HIV-exposed newborns and mothers are mostly integrated with the regular immunization schedule of home visit-based national programs (Chi *et al.*, 2013). These examples give us more contexts to consider regarding the application of IHCs by broadening the target population, where an existing immunization campaign permits.

This review once again emphasizes the importance of the schools as outreach or campaign site evidenced in immunization to reach adolescents and young people with health interventions. The widespread and continuing use of school-based programs is a testament to their success. It can be discussed in light of Human Papilloma Virus (HPV), vaccination of which most commonly targets adolescent girls in their 5th-6th grade using school-based platforms; seen in the Southeast Asian region in Bhutan, Malaysia and Thailand, South Africa within the Sub-Saharan African region and also (Amponsah-Dacosta *et al.*, 2022), High Income-country such as—Australia (Tsai *et al.*, 2018; Tsu *et al.*, 2021; World Health Organization, 2017; Jacob *et al.*, 2010; Al-Naggar *et al.*, 2012). While the school-based campaigns are commendable and the examples to be adopted by other LMICs; this translates into scope for integration of adolescent health services with existing immunization campaigns (i.e. HPV vaccine). A study conducted in two states of India (Andhra Pradesh and Gujarat) recommends HPV vaccines to be given through either routine immunization or a campaign that is based in a school for the implementation site, to achieve greater success (Jacob *et al.*, 2010). On the same note, broadening the target population may work as well in school-based platforms, as it can help include more stakeholders and beneficiaries, such as for the previous example of HPV—the school staff and parents besides just the female students, and services targeting them such as—sexual and reproductive health services and education, nutrition and hygiene-related interventions, etc. This can be supported by the example of a -country, Togo that reports such integration to be well accepted by all the stakeholders (Engel *et al.*, 2022). By involving the school authorities to support logistics and human resources, IHCs can leverage the school communities to raise awareness, improve community acceptance and uptake of the services, and build ownership of the campaigns; in one word, make a higher chance of implementation to succeed. In current times of COVID-19 vaccination for school-going children and adolescents, the possibility of integration where applicable and feasible should be considered with much scrutiny at policy levels.

### Lack of scientific research on government programs: a major gap

Despite major efforts by the governments in many countries to integrate campaigns, we suspect that these efforts are not always reflected in academic articles. This is observable as was in the case of Bangladesh, where the number of grey articles surpasses the number of scholarly articles (7 to 1 for Bangladesh) on the same topic, and likely applies to other countries as well. Examination of the Co-Delivered Campaign Calendar hosted on the Health Campaign Effectiveness Coalition website indicates hundreds of active IHCs implemented globally, which is significantly higher than what this scoping review revealed (The Task Force for Global Health, 2022). LMICs, especially those with poor representation among the included documents of this study, bear closer examination in future studies as it is evident that there is a large gap between the availability of scientific publications and the actual practice of IHCs. This factor is indicative of a gap in knowledge transfer from the field to the academic world and demands that more review works would be conducted in the future, sharing knowledge of implementation strategies and practical barriers from diverse health systems. Moreover, more research generating service delivery evidences, such as- coverage, efficiency, cost and cost-effectiveness data will be crucial to incorporate insights into policy and programs (Morgan *et al.*, 2022).

## Limitations

This review has several key limitations. Firstly, the review examined all LMICs only, whether Least-developed economies would have IHCs in place was outside the scope of this review. We recommend future reviews to explore this possibility. Documents published in Bengali and English were considered only due to the reviewers’ composition, leaving a huge limitation of the language barrier when including relevant grey literature most likely to be in various local languages. Similarly, grey literature from the national governments and local organizations of each LMICs could not be examined (55 countries in total). We assume that richer data exist in such sources documenting the countries’ progress with IHC, hence we urge scoping of reviews of grey literature from LICs and LMICs on the topic. The concept of an ‘integrated health campaign’ as a specific, discrete implementation strategy is relatively novel. Countries may utilize different terminologies for the same concept locally and was not possible to capture within the scope of this review. We tried to minimize this limitation for English documents with a broad range of synonyms in our search provision (Table 2), and once again recommend future document reviews of grey literature to bring out local insights in this area of implementation science. Lastly, we sought for reviewing various implementation parameters (effectiveness, feasibility, access, etc.), but a dearth of information from the articles prevented us in this regard. We adjust our understanding to a need for more specific review questions and appropriate search terms to overcome this issue. We only include reported effectiveness/ coverage in the included documents with our search provisions and analyse and present them in narratives to overcome this issue.

## Conclusion

An integrated approach in immunization service delivery was found to successfully increase coverage using limited resources and finances, even in remote regions, as well as increasing community acceptance, reaching a specific target population, and increasing the impact of the given intervention. Although the studies in our review covered the extent of coverage and the statistical successes of integration in detail, there is little information on the implementation parameters of integration. This identified gap in research on the implementation methods of integration and the undeniable necessity of IHCs in the context of LMICs makes it crucial that more implementation research on IHCs is carried out.

## Data Availability

All data used and/or analysed in the review were logged and are available from the corresponding author upon reasonable request.

## Reflexivity statement

The authors and reviewers list include seven females and one male. They range from various levels of seniority—early, mid and senior level researchers, also program-based professionals. Three of the authors come from a clinical health background, while one each from architecture, cell biology and behavioural science. Except two authors, all have varying experience, mostly advanced, of working in implementation research, covering topics such as—maternal and child health, quality of care, immunization, research methodologies etc. Three of the authors had prior experience of conducting a scoping review, with one to possess extensive experience. The authors are based in mostly Bangladesh, as well as USA.

## Appendix

**Table A1.** Key search terms for PubMed

Sl.	Key word ID	Key word	Remarks	Search results
1	PM_1	((Integrated health campaign OR Integrated health service OR Integrated health program OR Integrated health project OR Integrated health policy OR Integrated health plan OR Integrated health initiative) AND (Immunization OR vaccination OR vaccines OR inoculation) AND (evolution OR practice OR challenges OR success factors OR effectiveness OR evaluation OR lessons learned) AND (Low middle income countries OR LMICs))	All LMICs combined	132
2	PM_2	((Integrated health campaign OR Integrated health service OR Integrated health program OR Integrated health project OR Integrated health policy OR Integrated health plan OR Integrated health initiative) AND (Immunization OR vaccination OR vaccines OR inoculation) AND (evolution OR practice OR challenges OR success factors OR effectiveness OR evaluation OR lessons learned) AND (Low middle income countries OR LMICs AND Sub-Saharan Africa))	Region—sub-Saharan Africa	28
3	PM_3	((Integrated health campaign OR Integrated health service OR Integrated health program OR Integrated health project OR Integrated health policy OR Integrated health plan OR Integrated health initiative) AND (Immunization OR vaccination OR vaccines OR inoculation) AND (evolution OR practice OR challenges OR success factors OR effectiveness OR evaluation OR lessons learned) AND (Low middle income countries OR LMICs AND South Asia))	Region—South Asia	5
4	PM_4	((Integrated health campaign OR Integrated health service OR Integrated health program OR Integrated health project OR Integrated health policy OR Integrated health plan OR Integrated health initiative) AND (Immunization OR vaccination OR vaccines OR inoculation) AND (evolution OR practice OR challenges OR success factors OR effectiveness OR evaluation OR lessons learned) AND (Bangladesh))	Bangladesh	59
5	PM_5	(Integrated health campaign OR Integrated health service OR Integrated health program OR Integrated health project OR Integrated health policy OR Integrated health plan OR Integrated health initiative) AND (Immunization OR vaccination OR vaccines OR inoculation) AND (evolution OR practice OR challenges OR success factors OR effectiveness OR evaluation OR lessons learned) AND (Algeria)	Algeria	7
6	PM_6	(Integrated health campaign OR Integrated health service OR Integrated health program OR Integrated health project OR Integrated health policy OR Integrated health plan OR Integrated health initiative) AND (Immunization OR vaccination OR vaccines OR inoculation) AND (evolution OR practice OR challenges OR success factors OR effectiveness OR evaluation OR lessons learned) AND (Angola)	Angola	9
7	PM_7	(Integrated health campaign OR Integrated health service OR Integrated health program OR Integrated health project OR Integrated health policy OR Integrated health plan OR Integrated health initiative) AND (Immunization OR vaccination OR vaccines OR inoculation) AND (evolution OR practice OR challenges OR success factors OR effectiveness OR evaluation OR lessons learned) AND (Belize)	Belize	1
8	PM_8	(Integrated health campaign OR Integrated health service OR Integrated health program OR Integrated health project OR Integrated health policy OR Integrated health plan OR Integrated health initiative) AND (Immunization OR vaccination OR vaccines OR inoculation) AND (evolution OR practice OR challenges OR success factors OR effectiveness OR evaluation OR lessons learned) AND (Benin)	Benin	15
9	PM_9	(Integrated health campaign OR Integrated health service OR Integrated health program OR Integrated health project OR Integrated health policy OR Integrated health plan OR Integrated health initiative) AND (Immunization OR vaccination OR vaccines OR inoculation) AND (evolution OR practice OR challenges OR success factors OR effectiveness OR evaluation OR lessons learned) AND (Bhutan)	Bhutan	2
10	PM_10	(Integrated health campaign OR Integrated health service OR Integrated health program OR Integrated health project OR Integrated health policy OR Integrated health plan OR Integrated health initiative) AND (Immunization OR vaccination OR vaccines OR inoculation) AND (evolution OR practice OR challenges OR success factors OR effectiveness OR evaluation OR lessons learned) AND (Bolivia)	Bolivia	7
11	PM_11	(Integrated health campaign OR Integrated health service OR Integrated health program OR Integrated health project OR Integrated health policy OR Integrated health plan OR Integrated health initiative) AND (Immunization OR vaccination OR vaccines OR inoculation) AND (evolution OR practice OR challenges OR success factors OR effectiveness OR evaluation OR lessons learned) AND (Cabo Verde OR Cape Verde)	Cabo Verde (Cape Verde)	1
12	PM_12	(Integrated health campaign OR Integrated health service OR Integrated health program OR Integrated health project OR Integrated health policy OR Integrated health plan OR Integrated health initiative) AND (Immunization OR vaccination OR vaccines OR inoculation) AND (evolution OR practice OR challenges OR success factors OR effectiveness OR evaluation OR lessons learned) AND (Cambodia OR Kampuchea)	Cambodia	21
13	PM_13	(Integrated health campaign OR Integrated health service OR Integrated health program OR Integrated health project OR Integrated health policy OR Integrated health plan OR Integrated health initiative) AND (Immunization OR vaccination OR vaccines OR inoculation) AND (evolution OR practice OR challenges OR success factors OR effectiveness OR evaluation OR lessons learned) AND (Cameroon)	Cameroon	28
14	PM_14	(Integrated health campaign OR Integrated health service OR Integrated health program OR Integrated health project OR Integrated health policy OR Integrated health plan OR Integrated health initiative) AND (Immunization OR vaccination OR vaccines OR inoculation) AND (evolution OR practice OR challenges OR success factors OR effectiveness OR evaluation OR lessons learned) AND (Comoros)	Comoros	0
15	PM_15	(Integrated health campaign OR Integrated health service OR Integrated health program OR Integrated health project OR Integrated health policy OR Integrated health plan OR Integrated health initiative) AND (Immunization OR vaccination OR vaccines OR inoculation) AND (evolution OR practice OR challenges OR success factors OR effectiveness OR evaluation OR lessons learned) AND (Congo Republic OR Congo-Brazzaville)	Congo Republic (Congo-Brazzaville)	63
16	PM_16	(Integrated health campaign OR Integrated health service OR Integrated health program OR Integrated health project OR Integrated health policy OR Integrated health plan OR Integrated health initiative) AND (Immunization OR vaccination OR vaccines OR inoculation) AND (evolution OR practice OR challenges OR success factors OR effectiveness OR evaluation OR lessons learned) AND (Côte d’Ivoire OR Ivory Coast)	Côte d’Ivoire (Ivory Coast)	13
17	PM_17	(Integrated health campaign OR Integrated health service OR Integrated health program OR Integrated health project OR Integrated health policy OR Integrated health plan OR Integrated health initiative) AND (Immunization OR vaccination OR vaccines OR inoculation) AND (evolution OR practice OR challenges OR success factors OR effectiveness OR evaluation OR lessons learned) AND (Djibouti)	Djibouti	0
18	PM_18	(Integrated health campaign OR Integrated health service OR Integrated health program OR Integrated health project OR Integrated health policy OR Integrated health plan OR Integrated health initiative) AND (Immunization OR vaccination OR vaccines OR inoculation) AND (evolution OR practice OR challenges OR success factors OR effectiveness OR evaluation OR lessons learned) AND (Egypt)	Egypt	32
19	PM_19	(Integrated health campaign OR Integrated health service OR Integrated health program OR Integrated health project OR Integrated health policy OR Integrated health plan OR Integrated health initiative) AND (Immunization OR vaccination OR vaccines OR inoculation) AND (evolution OR practice OR challenges OR success factors OR effectiveness OR evaluation OR lessons learned) AND (El Salvador)	El Salvador	4
20	PM_20	(Integrated health campaign OR Integrated health service OR Integrated health program OR Integrated health project OR Integrated health policy OR Integrated health plan OR Integrated health initiative) AND (Immunization OR vaccination OR vaccines OR inoculation) AND (evolution OR practice OR challenges OR success factors OR effectiveness OR evaluation OR lessons learned) AND (Eswatini OR Swaziland)	Eswatini (Swaziland)	5
21	PM_21	(Integrated health campaign OR Integrated health service OR Integrated health program OR Integrated health project OR Integrated health policy OR Integrated health plan OR Integrated health initiative) AND (Immunization OR vaccination OR vaccines OR inoculation) AND (evolution OR practice OR challenges OR success factors OR effectiveness OR evaluation OR lessons learned) AND (Ghana)	Ghana	49
22	PM_22	(Integrated health campaign OR Integrated health service OR Integrated health program OR Integrated health project OR Integrated health policy OR Integrated health plan OR Integrated health initiative) AND (Immunization OR vaccination OR vaccines OR inoculation) AND (evolution OR practice OR challenges OR success factors OR effectiveness OR evaluation OR lessons learned) AND (Haiti)	Haiti	11
23	PM_23	(Integrated health campaign OR Integrated health service OR Integrated health program OR Integrated health project OR Integrated health policy OR Integrated health plan OR Integrated health initiative) AND (Immunization OR vaccination OR vaccines OR inoculation) AND (evolution OR practice OR challenges OR success factors OR effectiveness OR evaluation OR lessons learned) AND (Honduras)	Honduras	10
24	PM_24	(Integrated health campaign OR Integrated health service OR Integrated health program OR Integrated health project OR Integrated health policy OR Integrated health plan OR Integrated health initiative) AND (Immunization OR vaccination OR vaccines OR inoculation) AND (evolution OR practice OR challenges OR success factors OR effectiveness OR evaluation OR lessons learned) AND (India)	India	264
25	PM_25	(Integrated health campaign OR Integrated health service OR Integrated health program OR Integrated health project OR Integrated health policy OR Integrated health plan OR Integrated health initiative) AND (Immunization OR vaccination OR vaccines OR inoculation) AND (evolution OR practice OR challenges OR success factors OR effectiveness OR evaluation OR lessons learned) AND (Indonesia)	Indonesia	39
26	PM_26	(Integrated health campaign OR Integrated health service OR Integrated health program OR Integrated health project OR Integrated health policy OR Integrated health plan OR Integrated health initiative) AND (Immunization OR vaccination OR vaccines OR inoculation) AND (evolution OR practice OR challenges OR success factors OR effectiveness OR evaluation OR lessons learned) AND (Iran OR Persia)	Iran	36
27	PM_27	(Integrated health campaign OR Integrated health service OR Integrated health program OR Integrated health project OR Integrated health policy OR Integrated health plan OR Integrated health initiative) AND (Immunization OR vaccination OR vaccines OR inoculation) AND (evolution OR practice OR challenges OR success factors OR effectiveness OR evaluation OR lessons learned) AND (Kenya)	Kenya	108
28	PM_28	(Integrated health campaign OR Integrated health service OR Integrated health program OR Integrated health project OR Integrated health policy OR Integrated health plan OR Integrated health initiative) AND (Immunization OR vaccination OR vaccines OR inoculation) AND (evolution OR practice OR challenges OR success factors OR effectiveness OR evaluation OR lessons learned) AND (Kiribati)	Kiribati	2
29	PM_29	(Integrated health campaign OR Integrated health service OR Integrated health program OR Integrated health project OR Integrated health policy OR Integrated health plan OR Integrated health initiative) AND (Immunization OR vaccination OR vaccines OR inoculation) AND (evolution OR practice OR challenges OR success factors OR effectiveness OR evaluation OR lessons learned) AND (Kyrgyz OR Kyrgyzstan OR Kirghizia)	Kyrgyz Republic (Kyrgyzstan)	7
30	PM_30	(Integrated health campaign OR Integrated health service OR Integrated health program OR Integrated health project OR Integrated health policy OR Integrated health plan OR Integrated health initiative) AND (Immunization OR vaccination OR vaccines OR inoculation) AND (evolution OR practice OR challenges OR success factors OR effectiveness OR evaluation OR lessons learned) AND (Lao OR Laos)	Lao (Laos)	16
31	PM_31	(Integrated health campaign OR Integrated health service OR Integrated health program OR Integrated health project OR Integrated health policy OR Integrated health plan OR Integrated health initiative) AND (Immunization OR vaccination OR vaccines OR inoculation) AND (evolution OR practice OR challenges OR success factors OR effectiveness OR evaluation OR lessons learned) AND (Lesotho)	Lesotho	3
32	PM_32	(Integrated health campaign OR Integrated health service OR Integrated health program OR Integrated health project OR Integrated health policy OR Integrated health plan OR Integrated health initiative) AND (Immunization OR vaccination OR vaccines OR inoculation) AND (evolution OR practice OR challenges OR success factors OR effectiveness OR evaluation OR lessons learned) AND (Mauritania)	Mauritania	2
33	PM_33	(Integrated health campaign OR Integrated health service OR Integrated health program OR Integrated health project OR Integrated health policy OR Integrated health plan OR Integrated health initiative) AND (Immunization OR vaccination OR vaccines OR inoculation) AND (evolution OR practice OR challenges OR success factors OR effectiveness OR evaluation OR lessons learned) AND (Micronesia OR FSM)	Micronesia	2
34	PM_34	(Integrated health campaign OR Integrated health service OR Integrated health program OR Integrated health project OR Integrated health policy OR Integrated health plan OR Integrated health initiative) AND (Immunization OR vaccination OR vaccines OR inoculation) AND (evolution OR practice OR challenges OR success factors OR effectiveness OR evaluation OR lessons learned) AND (Mongolia)	Mongolia	8
35	PM_35	(Integrated health campaign OR Integrated health service OR Integrated health program OR Integrated health project OR Integrated health policy OR Integrated health plan OR Integrated health initiative) AND (Immunization OR vaccination OR vaccines OR inoculation) AND (evolution OR practice OR challenges OR success factors OR effectiveness OR evaluation OR lessons learned) AND (Morocco)	Morocco	11
36	PM_36	(Integrated health campaign OR Integrated health service OR Integrated health program OR Integrated health project OR Integrated health policy OR Integrated health plan OR Integrated health initiative) AND (Immunization OR vaccination OR vaccines OR inoculation) AND (evolution OR practice OR challenges OR success factors OR effectiveness OR evaluation OR lessons learned) AND (Myanmar OR Burma)	Myanmar	11
37	PM_37	(Integrated health campaign OR Integrated health service OR Integrated health program OR Integrated health project OR Integrated health policy OR Integrated health plan OR Integrated health initiative) AND (Immunization OR vaccination OR vaccines OR inoculation) AND (evolution OR practice OR challenges OR success factors OR effectiveness OR evaluation OR lessons learned) AND (Nepal)	Nepal	23
38	PM_38	(Integrated health campaign OR Integrated health service OR Integrated health program OR Integrated health project OR Integrated health policy OR Integrated health plan OR Integrated health initiative) AND (Immunization OR vaccination OR vaccines OR inoculation) AND (evolution OR practice OR challenges OR success factors OR effectiveness OR evaluation OR lessons learned) AND (Nicaragua)	Nicaragua	8
39	PM_39	(Integrated health campaign OR Integrated health service OR Integrated health program OR Integrated health project OR Integrated health policy OR Integrated health plan OR Integrated health initiative) AND (Immunization OR vaccination OR vaccines OR inoculation) AND (evolution OR practice OR challenges OR success factors OR effectiveness OR evaluation OR lessons learned) AND (Nigeria)	Nigeria	87
40	PM_40	(Integrated health campaign OR Integrated health service OR Integrated health program OR Integrated health project OR Integrated health policy OR Integrated health plan OR Integrated health initiative) AND (Immunization OR vaccination OR vaccines OR inoculation) AND (evolution OR practice OR challenges OR success factors OR effectiveness OR evaluation OR lessons learned) AND (Pakistan)	Pakistan	41
41	PM_41	(Integrated health campaign OR Integrated health service OR Integrated health program OR Integrated health project OR Integrated health policy OR Integrated health plan OR Integrated health initiative) AND (Immunization OR vaccination OR vaccines OR inoculation) AND (evolution OR practice OR challenges OR success factors OR effectiveness OR evaluation OR lessons learned) AND (Papua New Guinea)	Papua New Guinea	16
42	PM_42	(Integrated health campaign OR Integrated health service OR Integrated health program OR Integrated health project OR Integrated health policy OR Integrated health plan OR Integrated health initiative) AND (Immunization OR vaccination OR vaccines OR inoculation) AND (evolution OR practice OR challenges OR success factors OR effectiveness OR evaluation OR lessons learned) AND (Philippines)	Philippines	25
43	PM_43	(Integrated health campaign OR Integrated health service OR Integrated health program OR Integrated health project OR Integrated health policy OR Integrated health plan OR Integrated health initiative) AND (Immunization OR vaccination OR vaccines OR inoculation) AND (evolution OR practice OR challenges OR success factors OR effectiveness OR evaluation OR lessons learned) AND (Samoa)	Samoa	1
44	PM_44	(Integrated health campaign OR Integrated health service OR Integrated health program OR Integrated health project OR Integrated health policy OR Integrated health plan OR Integrated health initiative) AND (Immunization OR vaccination OR vaccines OR inoculation) AND (evolution OR practice OR challenges OR success factors OR effectiveness OR evaluation OR lessons learned) AND (São Tomé and Príncipe OR Saint Thomas and Prince)	São Tomé and Príncipe	4
45	PM_45	(Integrated health campaign OR Integrated health service OR Integrated health program OR Integrated health project OR Integrated health policy OR Integrated health plan OR Integrated health initiative) AND (Immunization OR vaccination OR vaccines OR inoculation) AND (evolution OR practice OR challenges OR success factors OR effectiveness OR evaluation OR lessons learned) AND (Senegal)	Senegal	38
46	PM_46	(Integrated health campaign OR Integrated health service OR Integrated health program OR Integrated health project OR Integrated health policy OR Integrated health plan OR Integrated health initiative) AND (Immunization OR vaccination OR vaccines OR inoculation) AND (evolution OR practice OR challenges OR success factors OR effectiveness OR evaluation OR lessons learned) AND (Solomon Islands)	Solomon Islands	11
47	PM_47	(Integrated health campaign OR Integrated health service OR Integrated health program OR Integrated health project OR Integrated health policy OR Integrated health plan OR Integrated health initiative) AND (Immunization OR vaccination OR vaccines OR inoculation) AND (evolution OR practice OR challenges OR success factors OR effectiveness OR evaluation OR lessons learned) AND (Sri Lanka)	Sri Lanka	6
48	PM_48	(Integrated health campaign OR Integrated health service OR Integrated health program OR Integrated health project OR Integrated health policy OR Integrated health plan OR Integrated health initiative) AND (Immunization OR vaccination OR vaccines OR inoculation) AND (evolution OR practice OR challenges OR success factors OR effectiveness OR evaluation OR lessons learned) AND (Tajikistan)	Tajikistan	1
49	PM_49	(Integrated health campaign OR Integrated health service OR Integrated health program OR Integrated health project OR Integrated health policy OR Integrated health plan OR Integrated health initiative) AND (Immunization OR vaccination OR vaccines OR inoculation) AND (evolution OR practice OR challenges OR success factors OR effectiveness OR evaluation OR lessons learned) AND (Tanzania)	Tanzania	74
50	PM_50	(Integrated health campaign OR Integrated health service OR Integrated health program OR Integrated health project OR Integrated health policy OR Integrated health plan OR Integrated health initiative) AND (Immunization OR vaccination OR vaccines OR inoculation) AND (evolution OR practice OR challenges OR success factors OR effectiveness OR evaluation OR lessons learned) AND (Timor-Leste OR East Timor)	Timor-Leste	5
51	PM_51	(Integrated health campaign OR Integrated health service OR Integrated health program OR Integrated health project OR Integrated health policy OR Integrated health plan OR Integrated health initiative) AND (Immunization OR vaccination OR vaccines OR inoculation) AND (evolution OR practice OR challenges OR success factors OR effectiveness OR evaluation OR lessons learned) AND (Tunisia)	Tunisia	12
52	PM_52	(Integrated health campaign OR Integrated health service OR Integrated health program OR Integrated health project OR Integrated health policy OR Integrated health plan OR Integrated health initiative) AND (Immunization OR vaccination OR vaccines OR inoculation) AND (evolution OR practice OR challenges OR success factors OR effectiveness OR evaluation OR lessons learned) AND (Ukraine)	Ukraine	12
53	PM_53	(Integrated health campaign OR Integrated health service OR Integrated health program OR Integrated health project OR Integrated health policy OR Integrated health plan OR Integrated health initiative) AND (Immunization OR vaccination OR vaccines OR inoculation) AND (evolution OR practice OR challenges OR success factors OR effectiveness OR evaluation OR lessons learned) AND (Uzbekistan)	Uzbekistan	1
54	PM_54	(Integrated health campaign OR Integrated health service OR Integrated health program OR Integrated health project OR Integrated health policy OR Integrated health plan OR Integrated health initiative) AND (Immunization OR vaccination OR vaccines OR inoculation) AND (evolution OR practice OR challenges OR success factors OR effectiveness OR evaluation OR lessons learned) AND (Vanuatu)	Vanuatu	0
55	PM_55	(Integrated health campaign OR Integrated health service OR Integrated health program OR Integrated health project OR Integrated health policy OR Integrated health plan OR Integrated health initiative) AND (Immunization OR vaccination OR vaccines OR inoculation) AND (evolution OR practice OR challenges OR success factors OR effectiveness OR evaluation OR lessons learned) AND (Vietnam)	Vietnam	43
56	PM_56	(Integrated health campaign OR Integrated health service OR Integrated health program OR Integrated health project OR Integrated health policy OR Integrated health plan OR Integrated health initiative) AND (Immunization OR vaccination OR vaccines OR inoculation) AND (evolution OR practice OR challenges OR success factors OR effectiveness OR evaluation OR lessons learned) AND (West Bank and Gaza OR Palestine OR Palestinian)	West Bank and Gaza (Palestinian Territories)	6
57	PM_57	(Integrated health campaign OR Integrated health service OR Integrated health program OR Integrated health project OR Integrated health policy OR Integrated health plan OR Integrated health initiative) AND (Immunization OR vaccination OR vaccines OR inoculation) AND (evolution OR practice OR challenges OR success factors OR effectiveness OR evaluation OR lessons learned) AND (Zambia)	Zambia	31
58	PM_58	(Integrated health campaign OR Integrated health service OR Integrated health program OR Integrated health project OR Integrated health policy OR Integrated health plan OR Integrated health initiative) AND (Immunization OR vaccination OR vaccines OR inoculation) AND (evolution OR practice OR challenges OR success factors OR effectiveness OR evaluation OR lessons learned) AND (Zimbabwe)	Zimbabwe	44

**Table A2.** PRISMA extension for Scoping Reviews (PRISMA-ScR) checklist

Section	Item	PRISMA-ScR checklist items	Reported
Title			
Title	1	Identify the report as a scoping review.	Yes
Abstract			
Structured summary	2	Provide a structured summary that includes (as applicable): background, objectives, eligibility criteria, sources of evidence, charting methods, results and conclusions that relate to the review questions and objectives.	Yes
Introduction			
Rationale	3	Describe the rationale for the review in the context of what is already known. Explain why the review questions/objectives lend themselves to a scoping review approach.	Yes
Objectives	4	Provide an explicit statement of the questions and objectives being addressed with reference to their key elements (e.g. population or participants, concepts and context) or other relevant key elements used to conceptualize the review questions and/or objectives.	Yes
Methods			
Protocol and registration	5	Indicate whether a review protocol exists; state if and where it can be accessed (e.g. a Web address); and if available, provide registration information, including the registration number.	Yes, concept note
Eligibility criteria	6	Specify characteristics of the sources of evidence used as eligibility criteria (e.g. years considered, language and publication status) and provide a rationale.	Yes
Information sources	7	Describe all information sources in the search (e.g. databases with dates of coverage and contact with authors to identify additional sources), as well as the date the most recent search was executed.	Yes
Search	8	Present the full electronic search strategy for at least 1 database, including any limits used, such that it could be repeated.	Yes
Selection of sources of evidence†	9	State the process for selecting sources of evidence (i.e. screening and eligibility) included in the scoping review.	Yes
Data charting process	10	Describe the methods of charting data from the included sources of evidence (e.g. calibrated forms or forms that have been tested by the team before their use, and whether data charting was done independently or in duplicate) and any processes for obtaining and confirming data from investigators.	Yes
Data items	11	List and define all variables for which data were sought and any assumptions and simplifications made.	Yes
Critical appraisal of individual sources of evidences	12	If done, provide a rationale for conducting a critical appraisal of included sources of evidence; describe the methods used and how this information was used in any data synthesis (if appropriate).	No
Synthesis of results	13	Describe the methods of handling and summarizing the data that were charted.	Yes
Results			
Selection of sources of evidence	14	Give numbers of sources of evidence screened, assessed for eligibility and included in the review, with reasons for exclusions at each stage, ideally using a flow diagram.	Yes
Characteristics of sources of evidence	15	For each source of evidence, present characteristics for which data were charted and provide the citations.	Yes
Critical appraisal within sources of evidence	16	If done, present data on critical appraisal of included sources of evidence (see item 12).	No
Results of individual sources of evidence	17	For each included source of evidence, present the relevant data that were charted that relate to the review questions and objectives.	Yes
Synthesis of results	18	Summarize and/or present the charting results as they relate to the review questions and objectives.	Yes
Discussion			
Summary of evidence	19	Summarize the main results (including an overview of concepts, themes and types of evidence available), link to the review questions and objectives and consider the relevance to key groups.	
Limitations	20	Discuss the limitations of the scoping review process.	Yes
Conclusions	21	Provide a general interpretation of the results with respect to the review questions and objectives, as well as potential implications and/or next steps.	Yes
Funding			
Funding	22	Describe sources of funding for the included sources of evidence, as well as sources of funding for the scoping review. Describe the role of the funders of the scoping review.	Yes

**Table:** Abbreviations

AIDS	Acquired Immunodeficiency Syndrome
ANC	Antenatal care
AVW	African vaccination week
BCG	Bacillus Calmette-Guérin
CDC	Centers for Disease Control and Prevention
CHD	Child health day
DGHS	Directorate General of Health Services
EPI	Expanded program on immunization
FP	Family planning
GVAP	Global vaccine action plan
HIV	Human immunodeficiency virus
HPV	Human papillomavirus
HR	Human resources
IHC	Integrated health campaign
ITN	Insecticide treated (bed)net
LMIC	Low- and middle-income country
MDA	Mass drug administration
MMR	Measles, mumps and rubella
MNCH	Maternal, neonatal and child health
MR	Measles and rubella
NID	National immunization day
NTD	Neglected tropical disease
NVAC	National vitamin A plus campaign
OPV	Oral polio vaccine
ORS	Oral rehydration solution
PCV	Pneumococcal vaccine
PI	Principal investigator
PRISMA	Preferred Reporting Items for Systematic Reviews and Meta-Analyses
SDG	Sustainable development goals
WASH	Water, sanitation and hygiene
WHO	World Health Organization
